# Mathematical modeling to assess health and economic impact of cardiovascular interventions and implementation strategies among people living with HIV: SAIA HTN

**DOI:** 10.1186/s43058-026-00887-1

**Published:** 2026-02-25

**Authors:** Akash Malhotra, Ana Olga Mocumbi, Maria Joana Coutinho, Maxinel Jeremias Filipe Chidácua, Amido Charama, Onei A. Uetela, Carmen Hazim, Isaias Ramiro, Kenneth Sherr, Sarah Gimbel, David Watkins

**Affiliations:** 1 Department of Global Health, University of Washington, Seattle, WA USA; 2 Universidade Eduardo Mondlane, Maputo, Mozambique; 3 Comite Para Saude de Moçambique, Maputo, Mozambique; 4 University of Washington School of Nursing, Seattle, WA USA

**Keywords:** Hypertension, HIV, Cardiovascular disease, Sub-Saharan Africa, Mozambique, Implementation strategy, Mathematical modeling, Decision-analytic model, Cost-effectiveness, Economic evaluation

## Abstract

**Background:**

Few economic evaluations distinguish between the cost and impact of evidence-based interventions and the strategies used to improve their implementation. This distinction is essential for understanding whether a strategy is cost-effective, why it works, and the resources required to replicate its success. The Systems Analysis and Improvement Approach Hypertension (SAIA-HTN) trial evaluated an implementation strategy (“SAIA”) designed to improve hypertension care among people living with HIV (PLHIV) in Mozambique. We developed a mathematical model to estimate the cost-effectiveness of both the evidence-based intervention (including hypertension screening, pharmacological treatment and follow up, and lifestyle modifications such as diet and exercise) and the SAIA implementation strategy.

**Methods:**

We constructed a decision-analytic, state-transition model that simulated cardiovascular risk, outcomes, and associated costs for PLHIV receiving hypertension care in Mozambique using a health systems perspective. Model inputs came from published epidemiological studies and primary data from the SAIA-HTN trial on intervention and implementation strategy effectiveness and costs. We estimated the incremental cost-effectiveness (willingness to pay $647/DALY averted, GDP per capita in Mozambique) of rolling out both components, compared to a “status quo” scenario where screening and treatment of hypertension remained at their current (very low) levels. Costs were reported in 2023 US dollars, and costs and outcomes were discounted at 3% over a ten-year time horizon.

**Results:**

Scaling up screening and pharmacological treatment of hypertension in Mozambique would have an incremental cost-effectiveness ratio (ICER) of around $212 per disability-adjusted life year (DALY) averted and cost an additional $4.61 per person per year. Incremental to the intervention, the SAIA implementation strategy would have an ICER of $44 per DALY averted and cost an additional $0.79 per person per year. The average reduction in ten-year cardiovascular risk would be 29.3% for the intervention and 40.3% if the SAIA implementation strategy were co-introduced.

**Conclusions:**

Our model is a tool for implementation scientists, policymakers, and researchers aiming to assess cardiovascular interventions and associated implementation strategies among PLHIV. Its application to SAIA-HTN suggests that this is a cost-effective strategy for improving hypertension care, but only in the presence of adequate blood pressure equipment, training, and medications. Our study shows how implementation strategies require a minimum threshold of health system readiness to generate meaningful health impact.

**Trial registration:**

ClinicalTrials.gov (NCT04088656).

**Supplementary Information:**

The online version contains supplementary material available at 10.1186/s43058-026-00887-1.

Contributions to the literature

- Studies rarely separate the cost and impact of evidence-based interventions from the strategies used to implement them. This study distinguishes both components using a decision-analytic modeling approach.
- The study demonstrates how economic evaluation can be embedded within implementation science to assess the value of a systems engineering–based implementation strategy (SAIA) alongside clinical hypertension care for people living with HIV.
- The findings show that implementation strategies can be highly cost-effective but require a minimum level of health system readiness to achieve impact.
- The framework offers a practical approach for evaluating and adapting implementation strategies in other diseases and low-resource settings.

## Background

Cardiovascular (CV) illness is the leading cause of morbidity and mortality amongst non-communicable diseases (NCDs) in Mozambique, accounting for over 40% of NCD deaths, and about 11% of death from all causes [1]. In 2021 in Mozambique, ischemic heart disease and stroke had prevalences of 0.84% and 0.73%, respectively, with an estimated 35,337 and 29,336 new cases that year [1]. Hypertension (HTN) is one of the major and modifiable risk factors for CV illness, especially amongst people living with HIV (PLHIV) [2]. In Mozambique, two in five adults are hypertensive, though only 14.5% know of their diagnosis, and only half of those are treated [3]. With the provision of antiretroviral therapy (ART), PLHIV are living longer and face a substantially higher risk of CV illness, estimated to be approximately 50% greater than in HIV-negative populations [4, 5]. As HIV has become a chronic, manageable condition, especially in countries with high ART coverage, the clinical and public health agenda has expanded to address emerging chronic comorbidities such as cardiovascular disease alongside continued HIV care [6]. Importantly, access to specialized interventions such as cardiac stents or thrombolytics remains very limited in Mozambique, underscoring the need for prevention through early detection and treatment of hypertension [7].

For patients with HIV and hypertension, co-management of both chronic conditions presents unique challenges and opportunities [8]. Existing resources like equipment and trained personnel are underutilized due to limited quality assurance across care points, resulting in suboptimal care integration [9]. For example, while clinics may have blood pressure measurement devices and staff trained to manage hypertension, these resources are not consistently integrated into routine HIV care [10]. This disconnect results in missed opportunities to identify and treat hypertension in HIV patients.

**Fig. 1:**
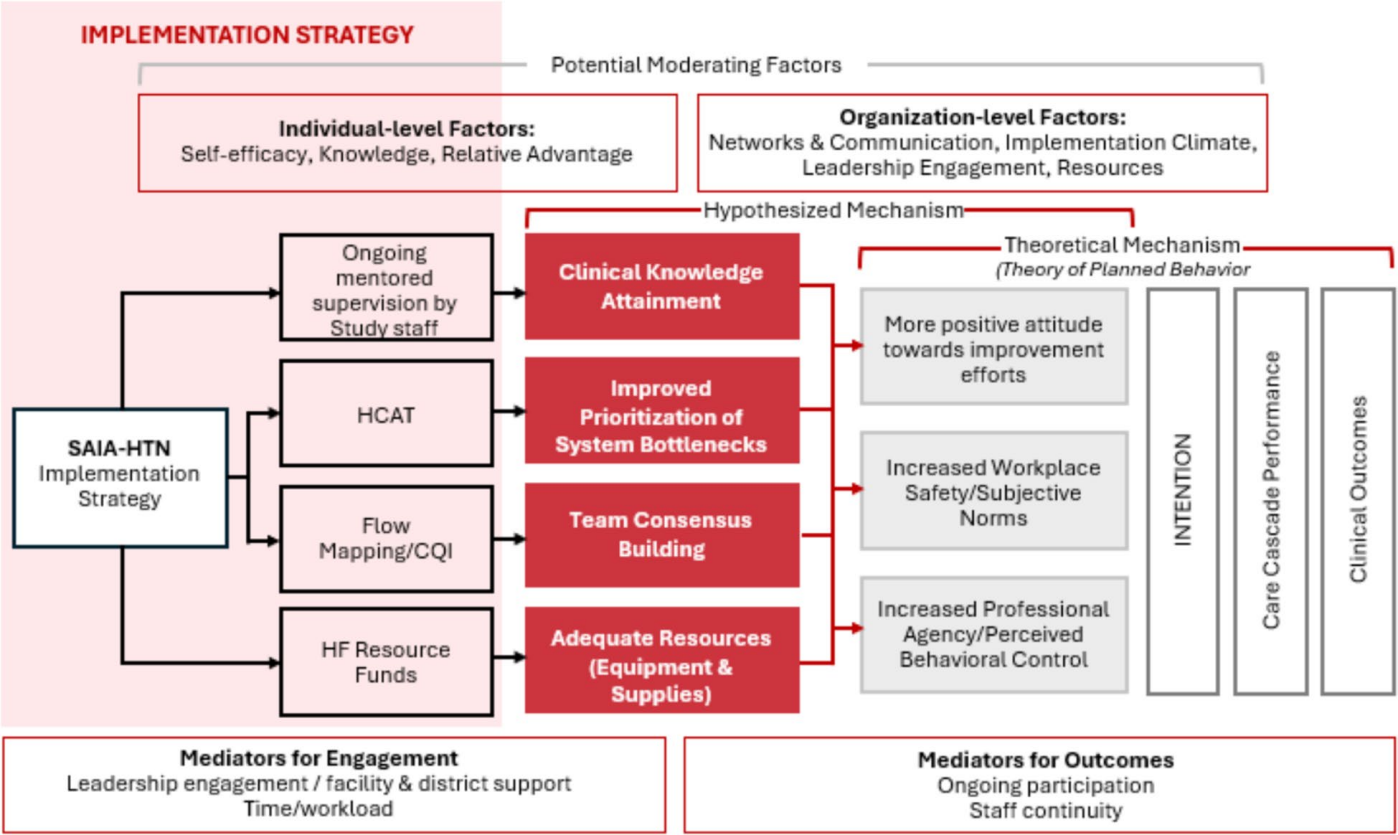
SAIA HTN logic model. Core SAIA components such as cascade analysis, process mapping, continuous quality improvement cycles, and mentored supervision, are mapped to proximal system-level changes, including improved identification of care bottlenecks, enhanced team coordination, clinical knowledge attainment, and adequate resources. These changes are expected to improve hypertension screening, treatment continuity, and blood pressure control, which ultimately reduce long-term CVD risk

The Systems Analysis and Improvement Approach (SAIA) provides a structured, scalable framework to standardize and improve quality across service points, ensuring consistent, integrated care along the HTN care cascade and ultimately improving health outcomes for individuals with HIV and HTN [9]. SAIA’s iterative, facility-based implementation strategy leverages process mapping and continuous quality improvement tools to address systemic inefficiencies. A simplified logic model for SAIA-HTN is presented in Fig. 1. The SAIA HTN trial, conducted in the Manica and Sofala provinces of Mozambique, between July 2020 and September 2023, demonstrated an increase in screening rates (37% at baseline vs. 99% post intervention roll out) and in process outcomes in the HTN care cascade such as treatment eligibility and medication pick up among PLHIV (Uetela O et al.: A: In press PLOS Medicine: Effectiveness of the systems analysis and improvement approach to optimize the hypertension care cascade for people living with HIV in central Mozambique: results from a hybrid type III cluster randomized trial, In press). Additionally, compared to the control arm, clients in the intervention arm were 2.22 times more likely to achieve hypertension control.

Few economic evaluations distinguish between the cost and impact of evidence-based interventions (EBIs) and the strategies used to improve their implementation [11]. This distinction is essential for understanding whether a strategy is cost-effective, why it works, and the resources required to replicate its success, thus helping understand the know-do gap from an economic standpoint. From a health economics perspective, the case for integrating hypertension screening and treatment into HIV care is strong. Models have demonstrated the cost-effectiveness of pharmacologic management of hypertension among PLHIV [12]. However, the feasibility and impact of real-world scale-up depend not just on clinical effectiveness or cost per pill, but on how these services are implemented. The presence of antihypertensive medications or blood pressure cuffs alone does not translate into health gains without accompanying strategies to support uptake, adherence, and system-level delivery improvements [13].

The objective of this analysis was twofold: to conduct a cost-effectiveness analysis of the SAIA-HTN trial in Mozambique, and to demonstrate how economic evaluation methods can be applied in an implementation science context to distinguish the contributions of evidence-based clinical interventions from those of implementation strategies. We developed a mathematical model to estimate the cost-effectiveness of both the evidence-based intervention (including hypertension screening, pharmacological treatment and follow-up, and lifestyle modifications such as diet and exercise) and the SAIA implementation strategy among people living with HIV. We use the SAIA-HTN trial as an applied case example to illustrate this modeling approach using real-world data. We introduce a flexible modeling framework that can be adapted by other settings to assess the value of implementation strategies using routine data and inform hypertension intervention strategies across diverse African healthcare settings. Our goal is to bridge the gap between economic evaluation and implementation science, to provide tools that help not only determine what works, but how to make it work better, and at what cost.

## Methods

### Study design

**Fig. 2:**
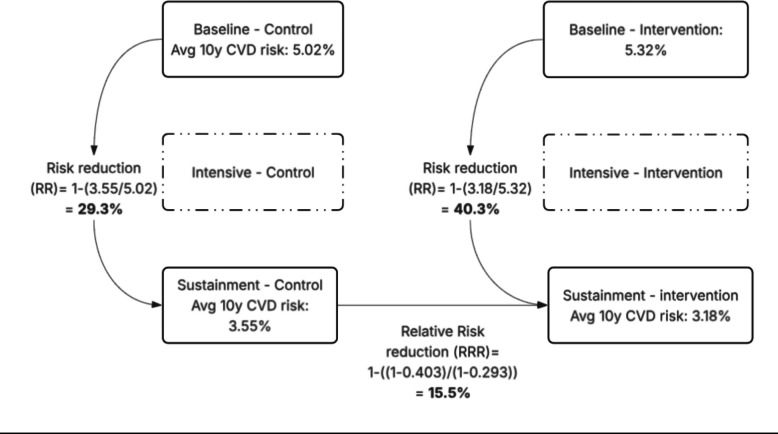
Change in 10-year CVD risk by arm and derivation of relative risk reduction. Solid boxes show baseline and sustainment mean 10-year cardiovascular risk for control and intervention arms; arrows indicate within-arm risk reduction and the cross-arm relative risk reduction (RRR). The intensive phase is shown for context

This cost-effectiveness analysis leverages a decision-analytical-model informed by the SAIA-HTN trial. The parent study design, protocol, and results have been reported elsewhere [9] (Uetela O et al.: A: In press PLOS Medicine: Effectiveness of the systems analysis and improvement approach to optimize the hypertension care cascade for people living with HIV in central Mozambique: results from a hybrid type III cluster randomized trial, In press). The trial compared HTN care among PLHIV against the SAIA implementation strategy across three sequential phases, i.e., baseline, intensive, and sustainment. The present analysis uses observed patient data and trial-based effectiveness estimates to simulate CV outcomes and costs across four modeled scenarios. A schematic of the two arms and three phases, along with the relative reduction in CV risk in each phase-arm combination is presented in Fig. 2.

### Model structure

**Fig. 3:**
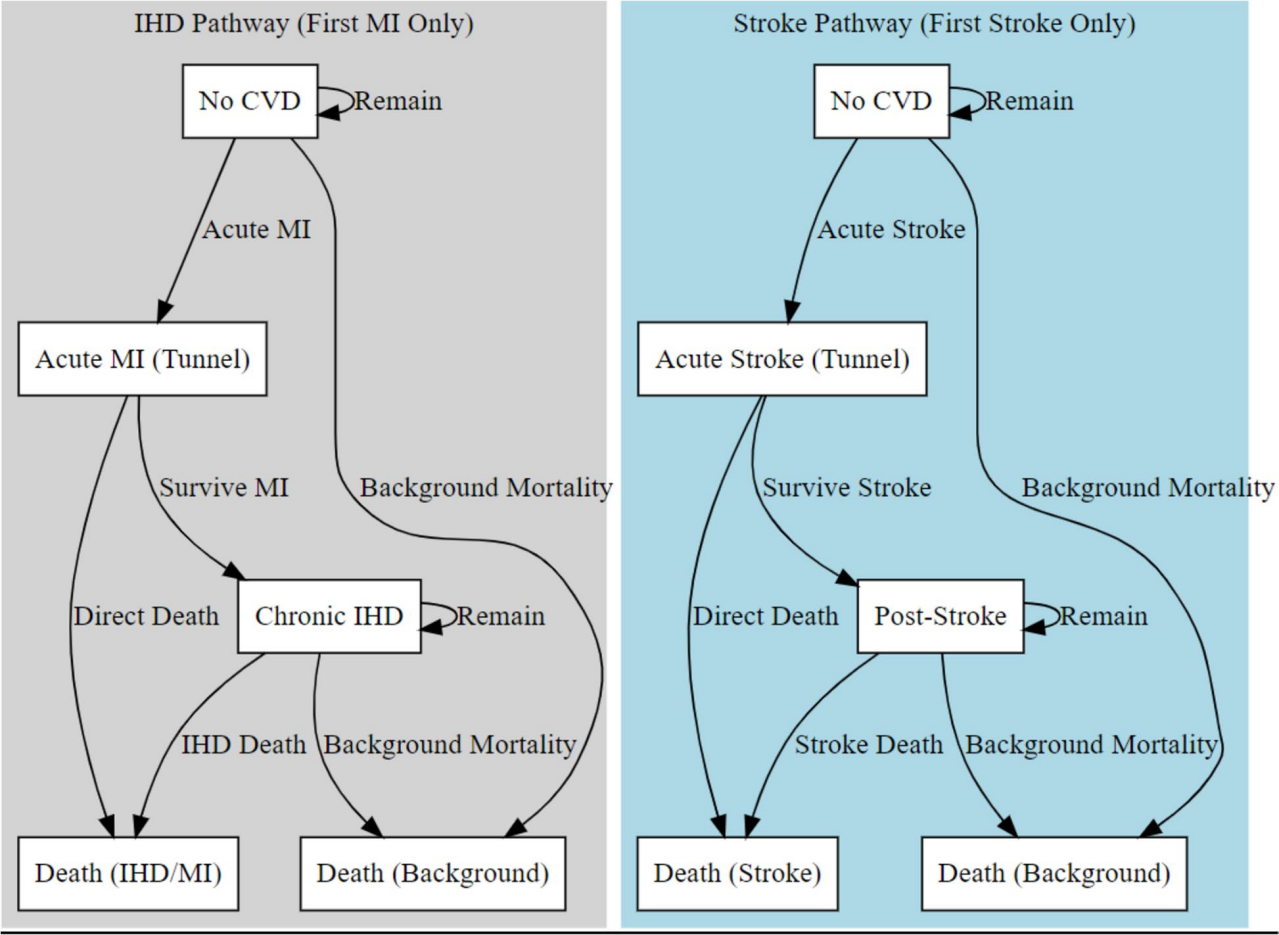
Decision-analytical model structure. Left panel: Ischemic Heart Disease/Myocardial Infarction (IHD/MI) pathway; right panel: stroke pathway. Individuals start in the no cardiovascular disease (CVD) state, may experience a first acute event (tunnel state), and either die acutely or survive to chronic IHD/post-stroke, with ongoing background and cause-specific mortality each monthly cycle. Only the first MI/stroke is modeled

The structure of the decision-analytic model was informed by standard cardiovascular disease modeling approaches and comprised two distinct pathways: one for ischemic heart disease (IHD) and another for stroke (Fig. 3). In each pathway, individuals begin in the “No CVD” state and may experience an initial acute event (acute myocardial infarction [MI] or acute stroke). Transitions between states occur in monthly cycles and are time- and risk-dependent. Patients could only experience one major CVD event (MI or stroke) during the simulation. Tunnel states for acute MI or stroke lasted one cycle. A small proportion of patients experiencing an event were assumed to access treatment. If they survive, patients who experienced an acute condition enter a chronic post-event state (chronic IHD or post-stroke), where they remain at risk for disease-specific mortality, background mortality, or remain stable.

A hybrid modeling structure was used. A subset of patients who appeared in all three phases of the SAIA-HTN trial formed the simulation cohort (S1 Table in Supplementary Text). Baseline cardiovascular risk for each patient was estimated using the WHO non-laboratory CVD risk charts [14].

Four scenarios were modeled (S1 Figure in Supplementary Text). The status quo, across both the intervention and control arm, assumed minimal to no screening or treatment for HTN and no change in cardiovascular risk. The sustainment phase in the control arm reflected potential improvements in hypertension care delivery. The fourth scenario included both the intervention and the SAIA strategy, with potentially additional gains in blood pressure control. Implementation costs were assigned based on facility, arm, and phase, with minimal “standard of care” costs in the baseline phase and additional labor and system strengthening costs attributed to the intensive and sustainment phases.

The model was implemented in R and simulated a 10-year time horizon from a health system perspective, with both costs (in 2023 US dollars) and health outcomes discounted at 3% annually [15].

### Data sources

Individual-level demographic and clinical data were extracted from the SAIA-HTN REDCap database, including age, sex, blood pressure (BP) measurements, body mass index (BMI), and smoking status. These characteristics were captured at every patient visit across all three study phases, i.e., baseline, intensive implementation, and sustainment, for both intervention and control arms. This information was used to estimate each patient’s 10-year cardiovascular risk as described previously. Observed reductions in systolic BP across the phases and arms were translated into changes in cardiovascular risk using evidence from a large meta-analysis, which found that a 5 mm Hg reduction in systolic BP reduced the risk of major cardiovascular events by approximately 10%, irrespective of prior CVD history or baseline BP level [16].

Transition probabilities, background mortality rates, disability weights, and life expectancy assumptions were sourced from the Global Burden of Disease (GBD) study, the WHO, and peer-reviewed literature [1, 17–21]. Mortality risk and life-years lost were applied depending on the specific age and sex of the patient using Mozambican life tables. Disability-adjusted life years (DALYs) were calculated by summing years of life lost (YLL) due to premature mortality and years lived with disability (YLD) from post-MI and post-stroke states. Model inputs are presented below in Table 1.

**Table 1:** Key model inputs

Parameter	Value	Source
Average 10-year cardiovascular risk reduction		
From control baseline to control sustainment (A)	29.3%	Primary data analysis
From intervention baseline to intervention sustainment (B)	40.3%	Primary data analysis
Relative risk reduction (1-(1-B)/(1-A))	15.5%	Primary data analysis
Relative proportion of CVD events that are MI	55%	[1]
Relative proportion of CVD events that are stroke	45%	[1]
Transition probabilities (Monthly)		
Acute MI to death	0.65	[18–20]
Chronic IHD to death	0.0034	[17]
Stroke to death	0.38	[17]
Post-stroke to death	0.0043	[17]
Background mortality	0.0018	[1]
Disability weights (Annual)		
Acute MI	0.439 (0.405, 0.477)	[17]
Chronic IHD	0.101 (0.093, 0.103)	[17]
Stroke	0.920 (0.782, 0.990)	[17]
Post-stroke	0.266 (0.228, 0.295)	[17]
Costs		
SAIA HTN trial expenditure		
Control arm	$355,448	Primary data analysis
Intervention arm	$468,126	Primary data analysis
Hypertension medication (per patient)		
Average annual per patient costs	$17.50	[22], calculated
% participants accessing treatment in baseline phase	2.1%	(Uetela O et al.: A: In press PLOS Medicine: Effectiveness of the systems analysis and improvement approach to optimize the hypertension care cascade for people living with HIV in central Mozambique: results from a hybrid type III cluster randomized trial, In press)
% participants accessing treatment in sustainment phase	8.2%	(Uetela O et al.: A: In press PLOS Medicine: Effectiveness of the systems analysis and improvement approach to optimize the hypertension care cascade for people living with HIV in central Mozambique: results from a hybrid type III cluster randomized trial, In press)
Cardiovascular outcomes		
Acute MI (one time)	$547 ($465, $629)	[23]
Chronic IHD (annual)	$12 ($10, $14)	[23]
Stroke (one time)	$317 ($265, $365)	[23]
Post-stroke (annual)	$14 ($12, $16)	[23]
CV illness treatment access for acute/emergency care	20% (0%, 80%)	Expert input

### Input costs

**Fig. 4:**
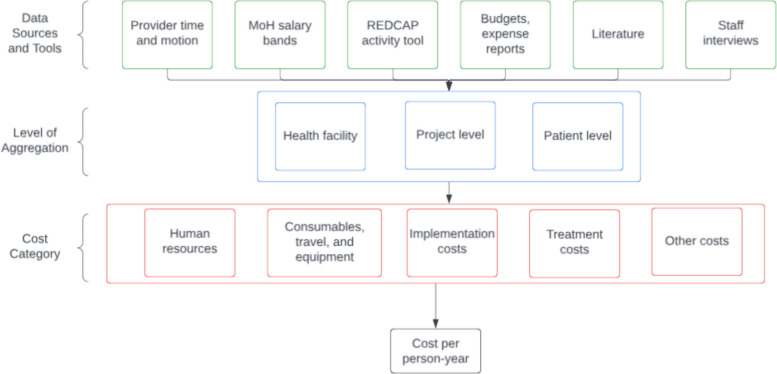
Costing framework and data flow. Primary inputs (provider time-and-motion, Ministry of Health salary bands, REDCap activity logs, budgets/expense reports, literature, staff interviews) are aggregated at health-facility, project, and patient levels and classified as human resources, consumables/travel/equipment, implementation, treatment, and other costs to derive cost per person-year

To estimate cost per patient-year of hypertension care and SAIA implementation, we combined both bottom-up and top-down costing approaches (Fig. 4). Primary cost data were collected during the trial using time and motion studies, provider-reported REDCap activity logs, Ministry of Health salary bands, facility and project budgets, and staff interviews. Costs were categorized as: provider salary, consumables/travel/equipment, SAIA implementation costs (e.g., coaching, dashboards, training), treatment costs (e.g., medication, clinical visits), and indirect/overhead costs. Costs were further tagged as research or program costs, with the latter used for the analysis [24].

Costs were aggregated at the facility, project, and patient levels, and then assigned to patients based on their utilization of services. Program costs which included human resources, consumables, travel, and other implementation costs were shared across all patients receiving screening in the respective arms, while treatment costs were assigned only to patients eligible for and initiated on HTN treatment. A proportion of the human resources costs were assigned for patient visits at baseline, to account for low levels of HTN screening at baseline. In the sustainment phase, total costs were converted to cost per person-year by dividing expenditures across each category by the number of patient-years observed per arm. Detailed breakdowns of inputs and cost calculations are available in the supplementary text (S2, S3, and S4 Tables).

### Cost-effectiveness analyses

We further classified and presented cost per person per year into three categories, i.e. direct costs of hypertension care (treatment of HTN and downstream CV illness, consumables, human resources); facility indirect costs, estimated using a markup of 7.4% for outpatient services and 27% for inpatient care [25]; and system strengthening costs, including the cost of implementing SAIA. Primary outcomes included incidence and mortality due to cardiovascular events, DALYs averted, total health system costs, and the incremental cost-effectiveness ratio (ICER). The primary outcomes have been modeled per 100,000 PLHIV seeking HTN care. Two ICERs were estimated based on the modeled scenarios (S1 Figure in supplementary text), one for the hypertension intervention (control sustainment vs. control baseline), and the other for the implementation strategy (intervention sustainment vs. control sustainment).

### Sensitivity analyses

We conducted both deterministic and probabilistic sensitivity analyses to assess how uncertainty in key inputs influenced model results.

For deterministic sensitivity analysis, we varied parameters one at a time. These included the cost of treating acute and chronic cardiovascular conditions, cost of antihypertensive medication, cost of implementing SAIA, the proportion of individuals receiving care after an event, the probability of death following MI or stroke, and the disability weights assigned to each cardiovascular state.

In addition, we implemented probabilistic sensitivity analysis (PSA) by drawing values from defined parameter distributions. We sampled cost and disability weight values using triangular and beta distributions, respectively, based on minimum, mean, and maximum values. Transition probabilities were also varied within plausible ranges.

For the intervention ICER, we also retained patient-level simulation in our model to reflect heterogeneity in baseline characteristics and disease risk. We simulated 334 individuals, for whom we had data across the three phases, under 100 distinct parameter sets (334 × 3 × 100 = > 100,000 draws). The risk reduction estimates in this simulation were informed from a larger dataset (*n* = 7,385). While the PSA captures parameter uncertainty (i.e., how model results vary due to inputs like cost or disability weight), the individual-level simulation allows us to account for heterogeneity across patients, including variation in baseline cardiovascular risk. This hybrid approach allowed us to represent both types of uncertainty in our cost-effectiveness results.

The study received ethical approval from Eduardo Mondlane University/Maputo Central Hospital and the University of Washington (CIBS FM&HCM/96/2018; STUDY00006694), and was registered on ClinicalTrials.gov (NCT04088656). This analysis fulfils the Consolidated Health Economic Evaluation Reporting Standards (CHEERS) checklist, which is attached as a supplementary file [26].

## Results

Detailed characteristics of the trial population and the subset used in our model are provided in the Supplementary Text (Sect. 2).

### Implementation cost

#### Per person per year

**Fig. 5:**
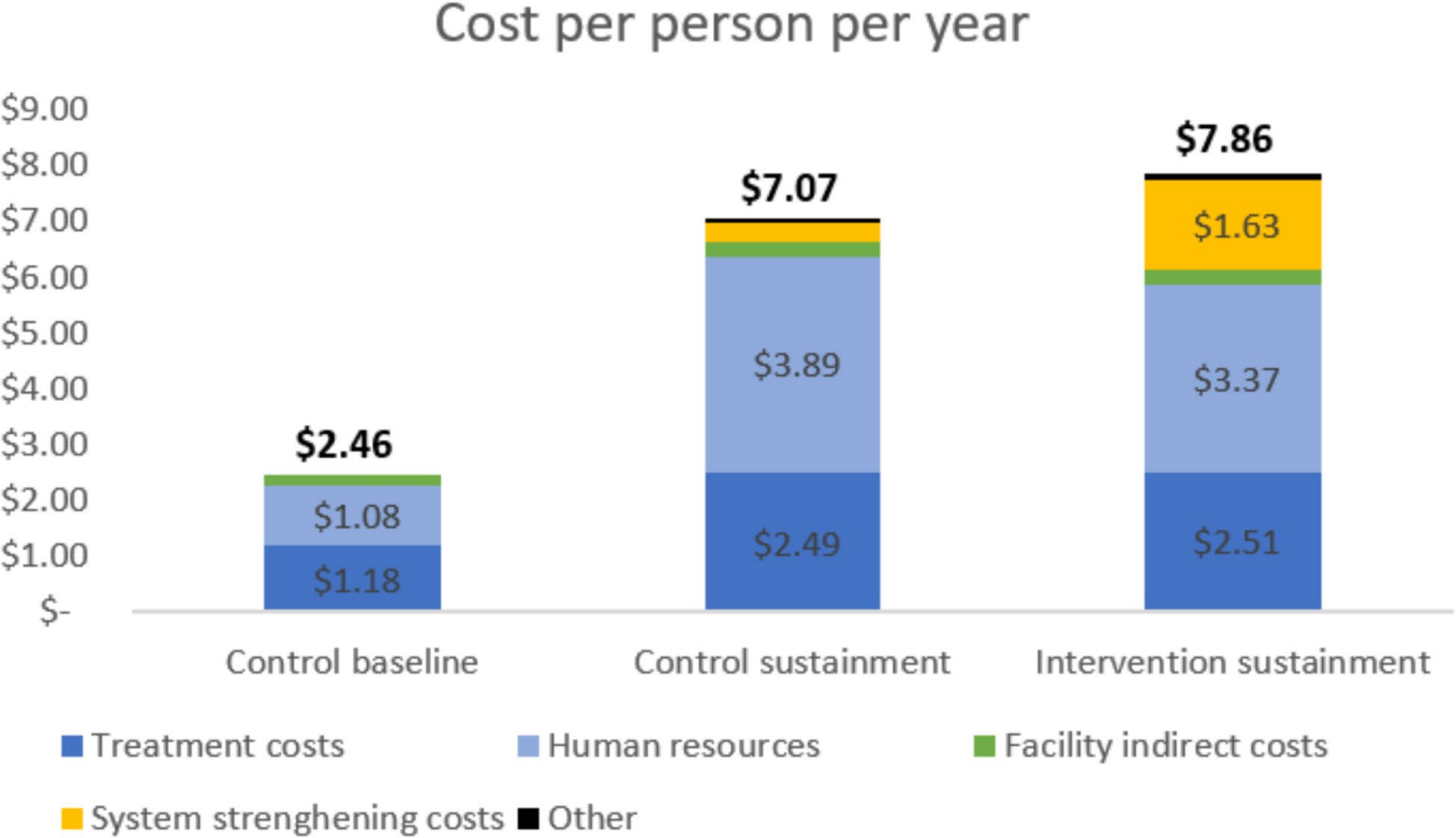
Cost per patient per year in control and intervention arms, by cost category. Each colored stack represents a unique cost category. The height of each stack is the contribution of that particular category to the overall cost per patient per year across both arms. The bars are stacked from bottom to top in the descending order of their relative contribution to the total cost in the control baseline. Treatment costs and human resources costs constitute direct healthcare costs. Treatment costs include costs of outpatient (HTN) and inpatient (MI and stroke) treatment, and related consumables. System strengthening costs including capacity building, meetings, and visits supporting the intervention and the SAIA implementation strategy. Labels are shown only for cost categories with values of at least $0.50 per patient-year

When expressed as average annual cost per person, the baseline phase of the control arm cost approximately $2.46 per patient-year (Fig. 5). This increased to $7.07 in the control sustainment phase, reflecting higher uptake of hypertension screening and treatment. In the intervention sustainment phase, per-patient costs increased further to $7.86, primarily due to the addition of SAIA program costs. At sustainment, human resource costs were slightly lower in the intervention arm because patients had fewer visits and hence lower apportioned costs for healthcare worker time. The incremental cost per patient-year of control sustainment compared with baseline was $4.61, and of intervention sustainment compared with control sustainment was $0.79.

#### Modeled costs and DALYs

Both the intervention and the SAIA implementation strategy substantially reduced cardiovascular events and deaths, while remaining highly cost-effective. Modeled results for 100,000 patients over the 10-year time horizon are presented in Table 2. Compared to the baseline phase of the control arm, the intervention reduced acute MI and stroke cases by 25% and 31%, and both MI and stroke deaths by 31% each. Compared to the sustainment phase of the control arm, the implementation strategy further reduced acute MI and stroke cases by 15.3% each, and deaths by 15% each. The intervention averted DALYs by 5.8%, leading to an ICER of $212 (95% UI: $48, $440) per DALY averted. The implementation strategy had an ICER of $44 (95% UI $42, $46) per DALY averted.

**Table 2:** 10-year model outcomes (per 100,000 PLHIV population)

	Control Baseline	Control Sustainment	Intervention Sustainment
Patient with acute MI	4389	3048	2582
Patients with stroke	3591	2494	2113
Deaths due to MI	2853	1981	1678
Deaths due to stroke	1365	948	803
Total DALYs	265,015	239,983	224,736
Total costs	$2.37 M	$6.16 M	$6.83 M
Intervention ICER	$212/DALY averted		-
Implementation strategy ICER	-	$44/DALY averted	

### Sensitivity analyses

**Fig. 6:**
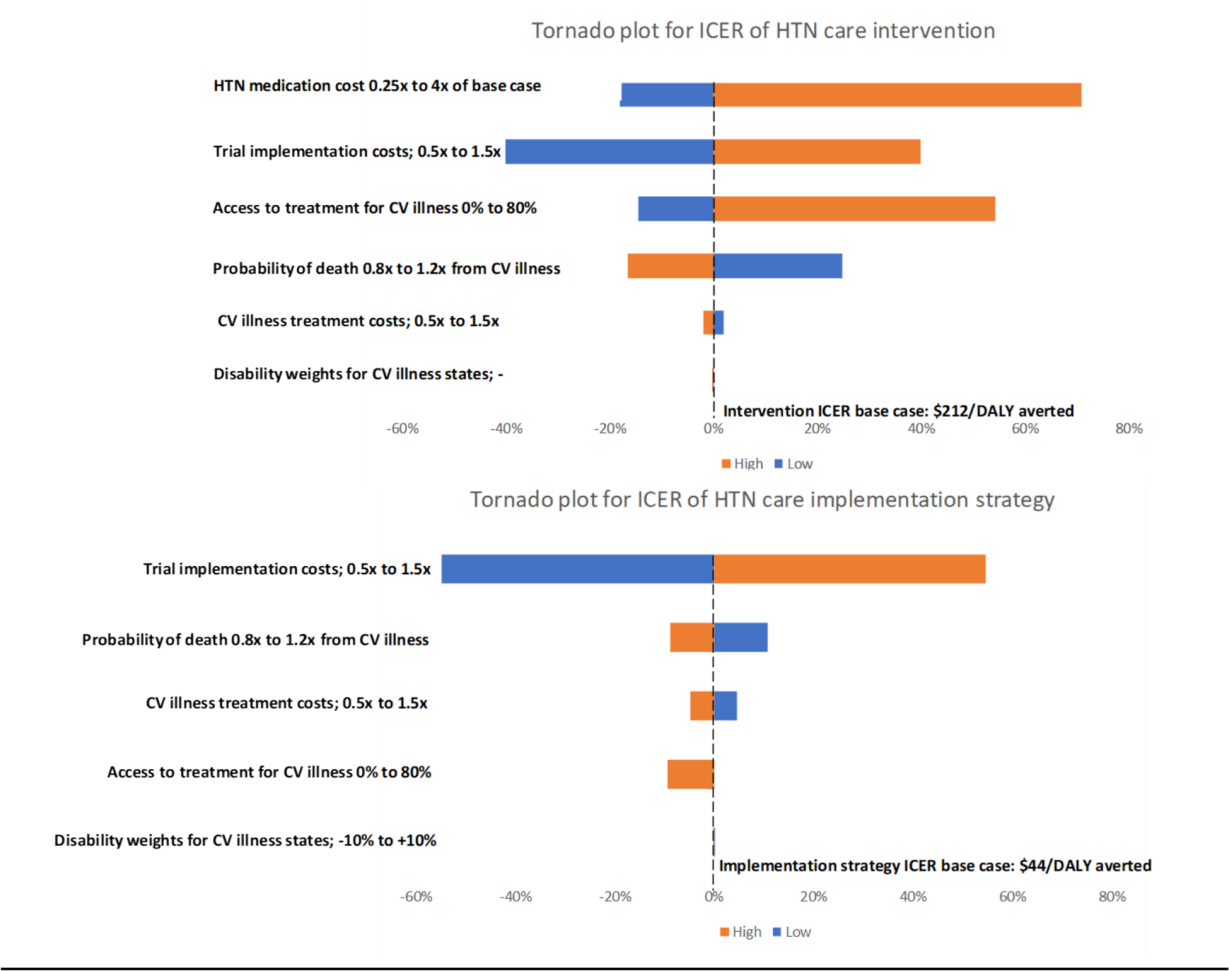
ICER Tornado plots. The reference vertical dashed line in both the plots represent the base case incremental cost effectiveness ratio (ICER) when all the parameters were set to their primary estimate values. The width of the bar indicates the relative change in the ICER with change in the value of the respective parameter. The blue bars indicate change in ICER when the value of the respective parameter is less than the primary estimate. The orange bars indicate change in ICER when the value of the respective parameter is more than the primary estimate. The drivers of cost are arranged in descending order of their influence on the ICER

One-way deterministic sensitivity analyses are shown in Fig. 6**.**

For the intervention ICER (control sustainment vs control baseline), results were most sensitive to variability in HTN medication costs, trial implementation costs, and access to treatment for CV illness. Variation in probability of death for acute CV conditions also had meaningful effects, while CV illness treatment costs and disability weights had limited impact. For the implementation strategy ICER (intervention sustainment vs control sustainment), the largest drivers of variation were trial implementation costs. Probability of death from CV illness, CV illness treatment costs, and access to CV illness treatment had little but meaningful impact, and as was the case with the intervention ICER, the disability weights has limited to no impact. Within a willingness to pay threshold of $647/DALY averted (Mozambique’s Gross Domestic Product or GDP per capita [27, 28]), the intervention and the implementation strategy remained cost-effective across all plausible parameter ranges.

**Fig. 7:**
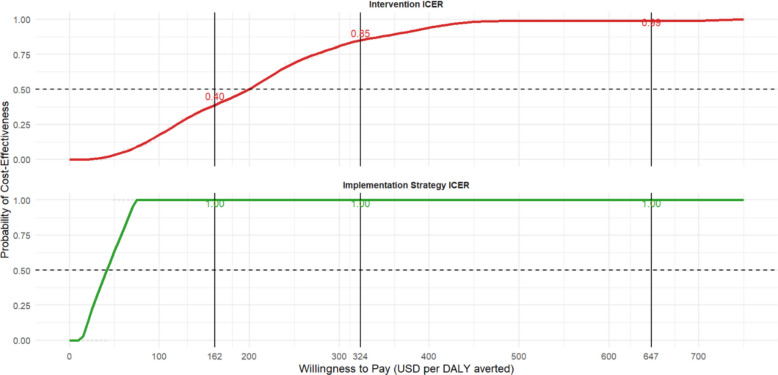
Cost-effectiveness acceptability curves. Cost-effectiveness acceptability curves for the SAIA-HTN intervention (Panel A in red) and the SAIA-HTN implementation strategy (Panel B in green). The intervention incremental cost-effectiveness ratio (ICER) is computed comparing the sustainment phase to the baseline phase in the control arm. The implementation strategy ICER is computed comparing the sustainment phase in the intervention arm to the control arm. Solid vertical lines represent alternative thresholds for evaluating cost-effectiveness, which include standard thresholds of 0.25x, 0.5x, and 1 × of the GDP per capita in Mozambique, $647, per DALY averted

In the probabilistic sensitivity analyses, both the intervention and the SAIA strategy were found to be consistently cost-effective, both being consistently more effective and more costly than the comparator across all simulation runs. At a willingness-to-pay threshold of $162.5 per DALY averted (0.25 × per capita GDP), the SAIA implementation strategy was cost-effective in 100% of simulations, while the intervention was cost-effective in 40%. At $325 (0.5 × per capita GDP), the probability of cost-effectiveness for the intervention was 85%, and at $647 (1 × per capita GDP), it exceeded 99%. The cost-effectiveness acceptability curve (CEAC) is shown in Fig. 7.

## Discussion

This analysis showed substantial improvements in 10-year cardiovascular risk outcomes among PLHIV receiving care for Hypertension under the SAIA-HTN trial in central Mozambique. Average annual cost per patient rose from $2.46 in the baseline phase to $7.07 in control sustainment, with a further increase to $7.86 in intervention sustainment due to added SAIA program costs. These investments translated into a 25–31% reduction in acute cardiovascular events and deaths over ten years. The intervention alone yielded an ICER of $212 per DALY averted, while adding SAIA reduced the ICER to just $44 per DALY averted, confirming that both strategies are highly cost-effective.

Strengthening basic hypertension care produced the largest reductions in cardiovascular risk, confirming the value of even modest improvements in diagnosis and treatment in high-prevalence settings. Adding SAIA on top of this foundation provided further health gains at low incremental cost, underscoring the role of implementation strategies in closing quality gaps. Taken together, these findings suggest that investments in both EBIs and the strategies to deliver them can work in tandem to generate health impact at scale.

Hypertension is highly prevalent in Mozambique, with two in five people (among those 25–64 years old) having high hypertension, yet awareness, treatment, and control rates remain low [3]. Several descriptive studies have emphasized the need for improved screening and sustained treatment. Economic evaluations of hypertension care in Mozambique are scarce. Evidence from other African settings shows that using existing health systems for population-level hypertension screening by community health workers, and treatment through integrated chronic care clinics, can reduce cardiovascular illness and is likely to be cost-effective [12]. Integrated six-month multi-month dispensing of HIV and hypertension medicines has also been shown to improve outcomes for patients already in care, reducing loss to follow-up and deaths by ensuring more reliable services [29]. These studies focused on the cost of medication and long-term health gains, with less attention to how services are delivered in practice. One of the few comparable economic evaluations is by Sando et al. (2020), who modeled the cost-effectiveness of integrating hypertension, diabetes, and high cholesterol screening and treatment into HIV care in Uganda and reported ICERs ranging from approximately $1,400 to $3,250 per DALY averted among older adults receiving ART (lower reduction in CVD risk compared to our intervention) [30]. While their analysis differs in scope and does not explicitly separate implementation strategies from clinical interventions, the magnitude of their cost-effectiveness estimates provides a relevant benchmark. Other studies in this area, such as evaluations of integrated HIV–NCD care in Malawi, have primarily focused on costs and clinical outcomes rather than cost-effectiveness [31].

By integrating individual-level trial data with a pragmatic modeling framework, we captured heterogeneity in baseline risk and simulated patient pathways under real-world conditions. We also explicitly separated the cost-effectiveness of the clinical intervention (hypertension treatment) from the cost-effectiveness of the implementation strategy (SAIA). This distinction has rarely been made in the literature, despite its policy relevance. By showing how delivery systems influence health outcomes, we add value by providing stronger evidence for how interventions can be implemented effectively in resource-limited health systems.

From a policy perspective, our findings suggest that investing in hypertension control should be a priority for Mozambique’s health system. Hypertension treatment is highly cost-effective, with major implications for reducing the burden of cardiovascular disease, which accounts for a large share of NCD mortality in the country. At the same time, SAIA provides an effective approach to improving quality of care where blood pressure control rates remain low. The SAIA strategy has been specified in prior work, which outlines its core components and intended pathways of effect and this analysis builds on that foundation by translating those system-level processes into health and economic outcomes [32]. The sequence of investments matters; Mozambique must first ensure that primary health centers can screen and treat hypertension reliably. Once this foundation is in place, SAIA can be deployed to strengthen quality of care and close implementation gaps.

Beyond Mozambique, other low- and middle-income countries facing similar challenges can adapt our model with local parameters, generating context-specific evidence to guide scale-up. The structure is flexible and can be applied across diverse settings. The underlying message is consistent, i.e., hypertension treatment is cost-effective, and quality improvement strategies like SAIA help ensure these benefits are realized.

From a research perspective, our study demonstrates the importance of evaluating both EBIs and the strategies used to deliver them. Implementation strategies often require additional resources, such as training, supervision, or workflow redesign, but their incremental costs are modest relative to the health gains they unlock. By modeling the contributions of both components, we show how routine care and quality improvement approaches can work in tandem. This provides a framework for future evaluations of implementation science interventions, which are often overlooked in economic analyses.

Further, it must be noted that the EBI reflects hypertension screening and treatment as delivered under existing standard-of-care conditions, which necessarily embed a baseline level of implementation activities and constraints. The SAIA strategy represents an explicit, theory-informed approach to strengthening and optimizing delivery beyond this baseline. Our broader motivation is to highlight that when implementation strategies are evaluated only in combination with clinical interventions, their incremental contribution may be obscured. By separating these components analytically, we aim to better characterize the added value of SAIA as an implementation strategy.

This study also has certain limitations. First, the disease structure was simplified. We modeled only one major CVD event (MI or stroke) per patient and assumed fixed probabilities for accessing acute and chronic care. We did not model hypertensive heart disease as an independent condition, even though it accounts for approximately 16% of cardiovascular deaths in Mozambique [1]. This omission, along with the lack of modeling of recurrent events, likely led to an underestimation of the total disease burden averted, correspondingly leading to an underestimation of the ICER. In addition, indirect societal costs such as lost productivity were not included, meaning our estimates may be conservative from a societal perspective. Secondly, cardiovascular risk was estimated using WHO non-laboratory risk charts parameterized for Eastern sub-Saharan Africa, which includes Mozambique and neighboring countries, and represent the closest available regionally relevant inputs. However, to our knowledge, these risk scores have not been formally validated among people living with HIV in Mozambique. Additionally, the generalizability of cost data and care patterns may be limited. Still, the model was designed to be flexible. Other settings can adapt it using their own parameters and choose between a simple cohort model or a more detailed patient-level simulation, depending on data availability. Lastly, the modeling cohort was relatively small (*n* = 334), restricted to patients who participated in all three phases of the trial. While this allowed for detailed tagging of costs and risk changes, it may not represent the broader population. We addressed this by applying risk reduction estimates from a larger dataset (*n* = 7,385), though assuming uniform effects across patients may not hold everywhere.

For future directions, rollout of SAIA is already underway and being evaluated through the SCALE-SAIA HTN study, a scaled up and scaled out expansion of the SAIA HTN trial [3]. This provides an opportunity to collect prospective data on costs including societal costs, data on quality improvements, on detailed health outcomes, and cost per cascade outcomes, enabling a more robust economic evaluation, including facility-level cost-effectiveness heterogeneity. This heterogeneity may even encompass variations in drug availability and workforce capacity. A cost-effectiveness analysis of SCALE-SAIA HTN will help refine parameter estimates and improve model accuracy. SCALE-SAIA HTN also creates an opportunity to examine equity-relevant dimensions, including whether SAIA reduces geographic and system-level disparities in access to hypertension care. Beyond Mozambique, the framework can be extended to other settings, adapted for general adult populations at risk of cardiovascular disease, and tailored to other NCD programs. These extensions will strengthen our understanding of how to maximize the value of both interventions and implementation strategies in diverse health systems.

## Conclusions

Our model is a tool for implementation scientists, policymakers, and researchers aiming to assess cardiovascular interventions and associated implementation strategies among PLHIV. Its application to SAIA-HTN in Mozambique suggests that this is a cost-effective strategy for improving hypertension care, but only in the presence of adequate blood pressure equipment, training, and medications. Our study illustrates how implementation strategies require a minimum threshold of health system readiness to generate meaningful health impact, and how economic evaluation methods can be applied to assess the added value of delivery strategies for chronic disease care in resource-limited settings.

## Supplementary Information

Supplementary Material 1.

## Data Availability

Costing data and model results generated or analysed during this study are included in this published article and/or the supplementary text. De-identified client data underlying the results reported in the parent study (which informed the effectiveness outcomes used in this article) and the respective data dictionary will be made available at the request of investigators whose proposed use of the data has been approved by an independent review committee identified for this purpose. Request submissions should be submitted to **saiastrategy@uw.edu**, and a data access agreement will need to be signed per the procedures of the University of Washington.

## Abbreviations

CV: Cardiovascular
NCD: Non-communicable diseases
HTN: Hypertension
PLHIV: People living with HIV
ART: Antiretroviral treatment
SAIA: Systems Analysis and Improvement Approach
IHD: Ischemic heart disease
MI: Myocardial infarction
BP: Blood pressure
BMI: Body mass index
GBD: Global Burden of Disease
DALYs: Disability-adjusted life years
YLL: Years of life lost
YLD: Years lived with disability
ICER: Incremental cost-effectiveness ratio
PSA: Probabilistic sensitivity analysis
CHEERS: Consolidated Health Economic Evaluation Reporting Standards
CEAC: Cost-effectiveness acceptability curve
GDP: Gross Domestic Product
