# Correction: Mathematical modeling to assess health and economic impact of cardiovascular interventions and implementation strategies among people living with HIV: SAIA HTN

**DOI:** 10.1186/s43058-026-01016-8

**Published:** 2026-06-15

**Authors:** Akash Malhotra, Ana Olga Mocumbi, Maria Joana Coutinho, Maxinel Jeremias Filipe Chidácua, Amido Charama, Onei A. Uetela, Carmen Hazim, Isaias Ramiro, Kenneth Sherr, Sarah Gimbel, David Watkins

**Affiliations:** 1 Department of Global Health, University of Washington, Seattle, WA USA; 2 Universidade Eduardo Mondlane, Maputo, Mozambique; 3 Comite Para Saude de Moçambique, Maputo, Mozambique; 4 University of Washington School of Nursing, Seattle, WA USA

**Correction: Implementation Science Communications (2026) 7:67**

10.1186/s43058-026-00887-1

In this article the Competing interests statement was incorrectly given as the authors declare no competing interests but should have been KS and SG are on the Editorial Board of Implementation Science Communications. The authors declare no further competing interests.

The original article has been corrected.

