# Supplementary material for: Mathematical modeling to assess health and economic impact of cardiovascular interventions and implementation strategies among people living with HIV: SAIA HTN
Source: Implement Sci Commun. 2026 Feb 25;7:67. doi: 10.1186/s43058-026-00887-1 (PMC13059167; doi:10.1186/s43058-026-00887-1)
Supplement: Supplementary file 1 — Supplementary Material 1. [file 43058_2026_887_MOESM1_ESM.docx]

**SUPPLEMENTARY TEXT**

Contents

[1. Additional Model Overview 1](#_Toc211522153)

[2. Model Population and CV Risk Reduction 2](#_Toc211522154)

[3. Other Key Model Parameters 4](#_Toc211522155)

[4. Uncertainty Analysis 8](#_Toc211522156)

[Supplementary Text References 9](#_Toc211522157)

## Additional Model Overview

**Mortality in the acute MI state**
To parameterize the probability of 30-day mortality after acute MI in Mozambique, where we assume that 80% of patients do not reach the hospital and only 20% are hospitalized, we drew on three complementary data sources. A clinical description of early MI mortality suggested that at least one-third of patients die before reaching hospital, and that 40–50% of those who arrive die rapidly upon admission[1]. This corresponds to an estimated non-hospitalized mortality of 75–83% and an overall mortality of roughly 63–76% under our assumed 80/20 split. Findings from another study, which reported 28-day case fatality rates across 29 populations, indicated a median of 50% overall mortality, with 25% among hospitalized patients. Back-calculating under the 80/20 split implied a non-hospitalized mortality of about 56%, with reported ranges suggesting overall mortality between 34% and 70%[2]. Finally, registry data from the Chinese Acute Stent Study (CASS) provided detailed case counts, reporting 79% 30-day mortality among non-hospitalized patients (239/302) and 19% among hospitalized patients (197/1,052), which translates into an overall 67% mortality under the 80/20 split[3].

Taken together, these sources converge on high early mortality when access to hospital care is limited. Using pathway-specific base values of approximately 79% for non-hospitalized patients and 25% for hospitalized patients, the overall 30-day mortality varies depending on the assumed distribution: about 79% if all patients are non-hospitalized (100/0 split), 65% under the Mozambique base case assumption of 80/20, and 37% if the majority are hospitalized (20/80 split). For modeling, we adopt a pooled base-case value of 65% overall 30-day mortality for Mozambique.

**Stroke events**
All stroke types were modeled together in a single acute stroke state, also a one-month tunnel. The case-fatality rate for stroke was taken directly from another modeling paper and applied uniformly[4]. Survivors entered the post-stroke state, where they faced elevated risk of death from stroke as well as background mortality.

**Relative distribution of first events (IHD vs. stroke)**
Once individuals were in the “No CVD” state, they faced a risk of experiencing their first cardiovascular event. When such an event occurred, 55% of the time it was modeled as an acute IHD event and 45% of the time as an acute stroke. This split was based on the relative incidence of stroke versus IHD in Mozambique in 2021, which reported 29,336 stroke cases and 35,337 IHD cases[5]. These numbers translate to just over half of all events being IHD [35,337/(35,337 + 29,336) ≈ 55%].

**Background mortality**
Background (non-CVD) mortality was applied to the No CVD, chronic IHD, and post-stroke states. Since the acute states (acute IHD and acute stroke) were one-month tunnel states, background mortality was not applied during those months. This ensured that deaths in the acute phase were attributed only to the cardiovascular event itself.

## Model Population and CV Risk Reduction

The SAIA-HTN trial enrolled 65,151 individuals receiving ambulatory HIV care across 16 public health facilities in central Mozambique[6]. For the purposes of modeling, we restricted to the subset of 7,385 patients who had complete data on age, sex, systolic blood pressure (SBP), body mass index (BMI), and smoking status, since these are the variables required to calculate 10-year cardiovascular disease (CVD) risk.

A further restricted subset of 334 individuals formed the individual-level simulation cohort. These were patients who appeared across all three phases of the trial (baseline, intensive, and sustainment), comprising 151 individuals in the control arm and 183 in the intervention arm. For each of these patients, baseline CVD risk was calculated, and the intervention effect was applied by scaling risk downward according to the arm-specific reductions derived from the larger 7,385-patient dataset. S1 Table below shows baseline characteristics for this analytic sample.

S1 Table. Patient characteristics at baseline (for simulation cohort)

|  | Intervention | Control |
| --- | --- | --- |
| Number of patients | 183 | 151 |
| Median age (IQR or 95% UI) | 48 (40 – 55) | 46 (37-55) |
| Female Sex (%) | 38% | 44% |
| Mean BMI (IQR or 95% UI) | 24.51 (21.72-26.67) | 25.11 (21.78 – 27.62) |
| Mean SBP (IQR or 95% UI) | 168.1 (154.0–179.5) | 166.6 (150.0–180.0) |
| Smokers (%) | 2.7% | 2.0% |
| 10-year CVD risk | 5.07 (3.00–7.00) | 5.02 (2.00–6.00) |

S1 Figure illustrates the cardiovascular risk reduction and the relative risk reduction between the arms.


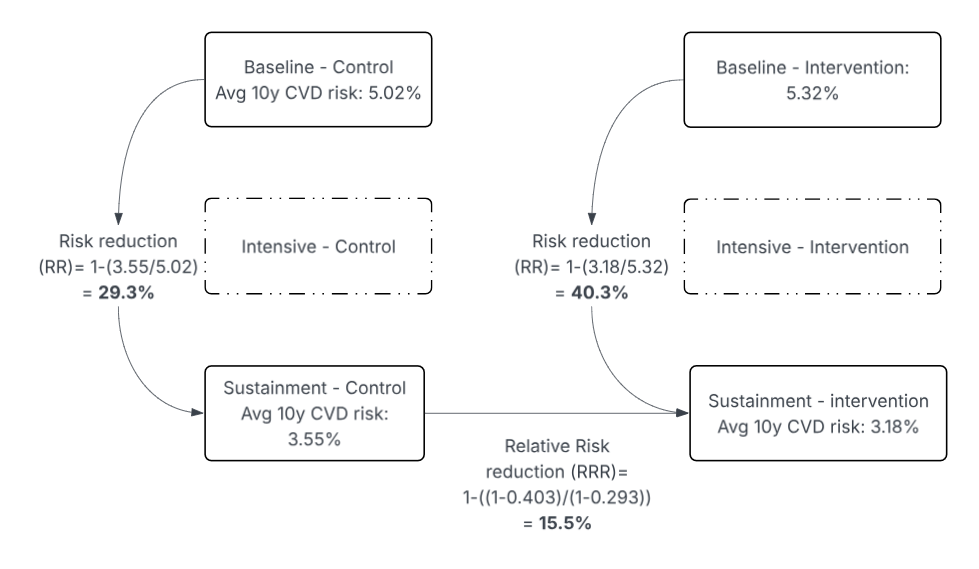


S1 Figure. Change in 10-year CVD risk by arm and derivation of relative risk reduction. Solid boxes show baseline and sustainment mean 10-year cardiovascular risk for control and intervention arms; arrows indicate within-arm risk reduction and the cross-arm relative risk reduction (RRR). The intensive phase is shown for context.

In the intermediary dataset with 7385 patients, at baseline, the mean 10-year CVD risk was 5.02% in the control arm and 5.32% in the intervention arm. By the sustainment phase, average risk declined to 3.55% in the control arm and 3.18% in the intervention arm (S1 Figure). This corresponded to a within-arm relative reduction of 29.3% in the control arm (1 – 3.55/5.02) and 40.3% in the intervention arm (1 – 3.18/5.32). When comparing across arms at sustainment, the intervention arm achieved an additional 15.5% relative risk reduction compared with the control arm.

## Other Key Model Parameters

**Costs**

In the main text, we report actual program costs and hypertension treatment costs for the baseline and sustainment phases, also presented in Figure 3. S2 Table below presents the program costs calculated from project expense sheets and activity-based costing tools.

| S2 Table. SAIA HTN Programmatic expenses* | | |  |
| --- | --- | --- | --- |
|  |  | Intervention | Control |
|  | Human resources | $ 297,502.07 | $ 297,502.07 |
|  | Other | $ 9,517.01 | $ 6,036.15 |
|  | Consumables | $ 49,614.84 | $ 44,113.43 |
|  | Support | $ 108,504.78 | $ 7,601.46 |
|  | Training | $ 2,987.80 | $ 195.15 |
|  | **TOTAL** | **$ 468,126.49** | **$ 355,448.25** |

These costs were first calculated per visit (with the number of visits later shown in S4 Table). The number of visits per patient in each arm was obtained from the main study: 4.25 visits per client in the intervention arm and 4.79 in the control arm. Because the trial lasted approximately 30 months, we converted these to effective annual visits by multiplying the total visits by 12/30 for each arm. We then added annualized activity costs, annual hypertension and CV treatment costs, and facility indirect costs, to obtain the cost per person-year across arms (as summarized in Figure 3 of the main text). Human resources costs in the baseline were determined by taking a proportion of the costs in the sustainment phase, i.e. the number of people seeking HTN treatment in the baseline vs sustainment arms (simplified assumption where staff involvement increased proportionally with patients seeking care when it comes to HTN access)

S3 Table. Breakdown of costs per person per year.

|  | Control baseline | Control sustainment | Intervention sustainment |
| --- | --- | --- | --- |
| Treatment costs | $ 1.18 | $ 2.49 | $ 2.51 |
| Human resources | $ 1.08 | $ 3.89 | $ 3.37 |
| Facility indirect costs | $ 0.20 | $ 0.23 | $ 0.24 |
| System strenghening costs | $ - | $ 0.38 | $ 1.63 |
| Other | $ - | $ 0.08 | $ 0.11 |

In the activity costing tool, we estimated the average meeting time for each SAIA visit, along with office supply and transportation costs associated with these meetings at the cluster level. For a post-trial scenario, we assumed each meeting would include one supervisor and one staff member, with monthly salaries of 50,000 MZN and 30,000 MZN, respectively, giving a combined monthly salary of 80,000 MZN. Assuming 22 working days per month and an 8-hour workday, this translated into an effective salary of 7.58 MZN per minute. The average meeting time (in minutes) was multiplied by the number of meetings and the effective salary per minute of attendees to calculate indirect HR costs per cluster.

Office supply costs were taken directly from reported expenses. For transportation, we assumed that in a post-trial scenario the Ministry of Health vehicle would be used for SAIA meetings. At a fuel price of 85 MZN per liter and a vehicle mileage of 11.11 km per liter, the effective cost was 7.65 MZN per km. Distances traveled for each visit were recorded and multiplied by mileage to generate transportation costs.

Indirect HR costs, office supplies, and transportation were then combined to calculate the effective activity cost per cluster. While some of these components may overlap with program expenses, our program expense sheets did not capture salaries of government staff or use of government vehicles. In addition, reporting costs by cluster helps capture the heterogeneity in SAIA implementation. S4 Table presents the effective activity costs per visit. The number of visits per cluster was taken from the main effectiveness study, and these costs were incorporated into the “system strengthening costs” category in Figure 3.

S4 Table. Activity costs per client visit

|  | Cluster | Arm | Phase | Visits | Cost (MZN) | Cost per visit (MZN) | Cost per visit (USD) |
| --- | --- | --- | --- | --- | --- | --- | --- |
| HR Buzi | 11 | int | baseline | 312 | - | - | - |
| CS Dondo | 12 | int | baseline | 1017 | - | - | - |
| CS Macurungo | 13 | con | baseline | 2934 | - | - | - |
| CS Mafambisse | 14 | con | baseline | 1080 | - | - | - |
| CS Mascarenhas | 15 | int | baseline | 1458 | - | - | - |
| CS Nhanconjo | 16 | int | baseline | 2713 | - | - | - |
| HR Nhamatanda | 17 | con | baseline | 992 | - | - | - |
| CS Ponte Gea | 18 | con | baseline | 1584 | - | - | - |
| HR Catandica | 21 | int | baseline | 467 | - | - | - |
| CS Eduardo Mondlane | 22 | int | baseline | 2900 | - | - | - |
| HD Manica | 23 | con | baseline | 1885 | - | - | - |
| CS Nhamahoanha | 24 | con | baseline | 1222 | - | - | - |
| CS 1 de Maio | 25 | int | baseline | 3056 | - | - | - |
| CS 7 de Abril | 26 | int | baseline | 2036 | - | - | - |
| CS Sussundenga | 27 | con | baseline | 1084 | - | - | - |
| CS Vanduzi | 28 | con | baseline | 888 | - | - | - |
| HR Buzi | 11 | int | intensive | 6756 | 135,477 | 20.05 | 0.31 |
| CS Dondo | 12 | int | intensive | 9168 | 90,791 | 9.90 | 0.15 |
| CS Macurungo | 13 | con | intensive | 27651 | 89,549 | 3.24 | 0.05 |
| CS Mafambisse | 14 | con | intensive | 10937 | 78,001 | 7.13 | 0.11 |
| CS Mascarenhas | 15 | int | intensive | 10347 | 36,239 | 3.50 | 0.05 |
| CS Nhanconjo | 16 | int | intensive | 17390 | 74,538 | 4.29 | 0.07 |
| HR Nhamatanda | 17 | con | intensive | 10331 | 86,496 | 8.37 | 0.13 |
| CS Ponte Gea | 18 | con | intensive | 7064 | 43,711 | 6.19 | 0.10 |
| HR Catandica | 21 | int | intensive | 5951 | 100,830 | 16.94 | 0.26 |
| CS Eduardo Mondlane | 22 | int | intensive | 17530 | 38,282 | 2.18 | 0.03 |
| HD Manica | 23 | con | intensive | 8145 | 70,456 | 8.65 | 0.14 |
| CS Nhamahoanha | 24 | con | intensive | 9762 | 17,436 | 1.79 | 0.03 |
| CS 1 de Maio | 25 | int | intensive | 13303 | 41,800 | 3.14 | 0.05 |
| CS 7 de Abril | 26 | int | intensive | 15385 | 12,559 | 0.82 | 0.01 |
| CS Sussundenga | 27 | con | intensive | 10278 | 33,982 | 3.31 | 0.05 |
| CS Vanduzi | 28 | con | intensive | 11463 | 27,740 | 2.42 | 0.04 |
| HR Buzi | 11 | int | sustainment | 3024 | 99,080 | 32.76 | 0.51 |
| CS Dondo | 12 | int | sustainment | 5694 | 87,475 | 15.36 | 0.24 |
| CS Macurungo | 13 | con | sustainment | 13858 | 55,251 | 3.99 | 0.06 |
| CS Mafambisse | 14 | con | sustainment | 3709 | 32,595 | 8.79 | 0.14 |
| CS Mascarenhas | 15 | int | sustainment | 4483 | 15,049 | 3.36 | 0.05 |
| CS Nhanconjo | 16 | int | sustainment | 7782 | 54,189 | 6.96 | 0.11 |
| HR Nhamatanda | 17 | con | sustainment | 2879 | 51,041 | 17.73 | 0.28 |
| CS Ponte Gea | 18 | con | sustainment | 3522 | 17,927 | 5.09 | 0.08 |
| HR Catandica | 21 | int | sustainment | 1513 | 62,111 | 41.05 | 0.64 |
| CS Eduardo Mondlane | 22 | int | sustainment | 5715 | 19,749 | 3.46 | 0.05 |
| HD Manica | 23 | con | sustainment | 3244 | 46,964 | 14.48 | 0.23 |
| CS Nhamahoanha | 24 | con | sustainment | 2978 | 25,671 | 8.62 | 0.13 |
| CS 1 de Maio | 25 | int | sustainment | 5170 | 20,907 | 4.04 | 0.06 |
| CS 7 de Abril | 26 | int | sustainment | 6547 | 20,397 | 3.12 | 0.05 |
| CS Sussundenga | 27 | con | sustainment | 4164 | 35,778 | 8.59 | 0.13 |
| CS Vanduzi | 28 | con | sustainment | 5093 | 33,951 | 6.67 | 0.10 |

From the literature, we estimated the per capita cost of hypertension medication in Mozambique to be $3.50 per year for adults aged 40–64[7]. Assuming 20% of the population is hypertensive, this translates to $17.50 per hypertensive patient per year. Using SAIA-HTN cascade data, we estimated that in the baseline phase, approximately 2.03% of participants were prescribed hypertension medication, while in the sustainment phase this proportion increased to 8.19%. Accordingly, we assumed an average annual per-patient cost of $0.40 in the baseline phase (17.5 × 2%) and $1.44 in the sustainment phase (17.5 × 8%).

**Data availability**

Spreadsheets with the raw data for these calculations, and the model code, are available in the GitHub repository: <https://github.com/akash210593/saiahtn> .

## Uncertainty Analysis

We incorporated parameter uncertainty through a probabilistic sensitivity analysis. Transition probabilities for mortality were varied within prespecified ranges, including acute deaths from myocardial infarction and stroke, deaths in the chronic IHD and post-stroke states, and background mortality. For each of these, values were drawn from beta distributions parameterized using the mean values and plausible bounds identified in the literature.

Program costs per patient were varied within observed ranges using triangular distributions defined by minimum, maximum, and mean values. Acute and chronic cardiovascular care costs were modeled in the same way, with triangular distributions reflecting their lower and upper bounds.

Disability weights for acute myocardial infarction, chronic IHD, acute stroke, and post-stroke sequelae were also varied. Beta distributions were constructed from the reported mean values and plausible ranges, ensuring that each draw remained bounded between zero and one.

A total of one hundred probabilistic draws were implemented. For each draw, costs, years lived with disability, and years of life lost were calculated over the ten-year horizon and then averaged by trial arm and phase. Results are reported as mean values with 95% uncertainty intervals, and incremental cost-effectiveness ratios were calculated for each probabilistic sample.

## Supplementary Text References

1. Mechanic O, Gavin M, Grossman S. Acute Myocardial Infarction [Internet]. Treasure Island, Florida: StatPearls Publishing; 2025. https://www.ncbi.nlm.nih.gov/books/NBK459269/

2. Chambless L, Keil U, Dobson A, Mähönen M, Kuulasmaa K, Rajakangas A-M, et al. Population Versus Clinical View of Case Fatality From Acute Coronary Heart Disease: Results From the WHO MONICA Project 1985–1990. Circulation. 1997;96:3849–59. https://doi.org/10.1161/01.CIR.96.11.3849

3. Davis KB, Alderman EL, Kosinski AS, Passamani E, Kennedy JW. Early mortality of acute myocardial infarction in patients with and without prior coronary revascularization surgery. A Coronary Artery Surgery Study Registry Study. Circulation. 1992;85:2100–9. https://doi.org/10.1161/01.CIR.85.6.2100

4. Subramanian S, Hilscher R, Gakunga R, Munoz B, Ogola E. Cost-effectiveness of risk stratified medication management for reducing premature cardiovascular mortality in Kenya. Chen S, editor. PLoS ONE. 2019;14:e0218256. https://doi.org/10.1371/journal.pone.0218256

5. Institute for Health Metrics and Evaluation (IHME), Seattle, WA, University of Washington. GBD Results [Internet]. 2024 [cited 2025 May 28]. https://vizhub.healthdata.org/gbd-results/. Accessed 28 May 2025

6. Uetela O, Mocumbi AO, Augusto O, Malhotra A, Brumwell A, Charama A. In press PLOS Medicine: Effectiveness of the systems analysis and improvement approach to optimize the hypertension care cascade for people living with HIV in central Mozambique: results from a hybrid type III cluster randomized trial.

7. Damasceno A, Padrão P, Silva-Matos C, Prista A, Azevedo A, Lunet N. Cardiovascular risk in Mozambique: who should be treated for hypertension? Journal of Hypertension. 2013;31:2348–55. https://doi.org/10.1097/HJH.0b013e3283656a0a
